# Supplementary material for: Assessment and Management of Maxillary Labial Frenum—A Scoping Review
Source: Diagnostics (Basel). 2024 Aug 6;14(16):1710. doi: 10.3390/diagnostics14161710 (PMC11352991; doi:10.3390/diagnostics14161710)
Supplement: Supplementary file 1 [file diagnostics-14-01710-s001.zip › diagnostics-3103820-supplementary.pdf]

Table S1. List of the online databases used, search strategies that were developed for each database and number of studies that were retrieved in each case.

Electronic Database	Search strategy	Ext end of search	No of hits
**MEDLINE**<br>Searched via PubMed on 08.04.2023<br>http://www.ncbi.nlm.nih.gov/pubmed/advanced	(("labial frenum*"[Title/Abstract] OR "labial frena"[Title/Abstract] OR "labial frenulum"[Title/Abstract] OR "labial frenula"[Title/Abstract] OR "upper lip frenum*"[Title/Abstract] OR ((("upper"[All Fields] OR "uppers"[All Fields]) AND ("lip"[MeSH Terms] OR "lip"[All Fields])) AND "frena"[Title/Abstract]) OR "upper lip frenulum*"[Title/Abstract] OR ((("upper"[All Fields] OR "uppers"[All Fields]) AND ("lip"[MeSH Terms] OR "lip"[All Fields])) AND "frenula"[Title/Abstract]) OR (("upper"[All Fields] OR "uppers"[All Fields]) AND "frenum*"[Title/Abstract]) OR (("upper"[All Fields] OR "uppers"[All Fields]) AND "frena"[Title/Abstract]) OR (("upper"[All Fields] OR "uppers"[All Fields]) AND "frenulum"[All Fields]) OR (("upper"[All Fields] OR "uppers"[All Fields]) AND "frenula"[Title/Abstract]) OR "maxillary frenum*"[Title/Abstract] OR (("maxilla"[MeSH Terms] OR "maxilla"[All Fields] OR "maxillary"[All Fields] OR "maxillaries"[All Fields] OR "maxillaris"[All Fields]) AND "frena"[Title/Abstract]) OR "maxillary frenulum*"[Title/Abstract] OR (("maxilla"[MeSH Terms] OR "maxilla"[All Fields] OR "maxillary"[All Fields] OR "maxillaries"[All Fields] OR "maxillaris"[All Fields]) AND "frenula"[Title/Abstract]) OR "maxillary labial frenum*"[Title/Abstract] OR (("maxilla"[MeSH Terms] OR "maxilla"[All Fields] OR "maxillary"[All Fields] OR "maxillaries"[All Fields] OR "maxillaris"[All Fields]) AND "labial frena"[Title/Abstract]) OR "mlf"[Title/Abstract]) AND ("diagnosis"[Title/Abstract] OR "assessment*"[Title/Abstract] OR "evaluation"[Title/Abstract] OR "management"[Title/Abstract] OR "frenotomy*"[Title/Abstract] OR "frenectomy*"[Title/Abstract] OR "referral*"[Title/Abstract] OR "health impact"[Title/Abstract])) AND ((humans[Filter]) AND (english[Filter]))	Searched by Title and Abstract; Filters: English Language, Humans	139
**Web of Science**<br>Searched via https://clarivate.com/web ofsciencegroup/solutions/ web-of-science/ on 08.04.2023	#1: ((((((((((((((((((TS=(labial frenum*)) OR TS=(labial frena)) OR TS=(labial frenulum)) OR TS=(labial frenula)) OR TS=(upper lip frenum)) OR TS=(upper lip frena)) OR TS=(upper lip frenulum)) OR TS=(upper lip frenula)) OR TS=(upper frenum)) OR TS=(upper frena)) OR TS=(upper frenulum)) OR TS=(upper frenula)) OR TS=(maxillary frenum)) OR TS=(maxillary frena)) OR TS=(maxillary frenulum)) OR TS=(maxillary frenula)) OR TS=(maxillary labial frenum)) OR TS=(maxillary labial frena)) OR TS=(maxillary labial frenulum)) OR TS=(maxillary labial frenula)) OR TS=(MLF)<br><br>#2: (((((((((TS=(diagnosis)) OR TS=(assessment*)) OR TS=(evaluation*)) OR TS=(management*)) OR TS=(frenotomy)) OR TS=(frenotomies)) OR TS=(frenectomy)) OR TS=(frenectomies)) OR TS=(referral*)) OR TS=(health impact)<br><br>#1 AND #2 and English (Languages)	Searched by "Topic" Filter: English language	213
**Cochrane Library**<br>Searched via http://onlinelibrary.wiley. Com/cochranelibrary/ on 08.04.2023	((labial frenum*) OR (labial frena) OR (labial frenulum) OR (labial frenula) OR (upper lip frenum*) OR (upper lip frena) OR (upper lip frenulum*) OR (upper lip frenula) OR (upper frenum*) OR (upper frena) OR (upper frenulum)* OR (upper frenula) OR (maxillary frenum*) OR (maxillary frena) OR (maxillary frenulum*) OR (maxillary frenula) OR (maxillary labial frenum*) OR (maxillary labial frena) OR (MLF*)) AND ((diagnosis) OR (assessment*) OR (evaluation) OR (management) OR (frenotomy*) OR (frenectomy*) OR (referral*) OR (health impact)) in Title Abstract Keyword	Searched by "Title Abstract Keyword" Filter: English language	99
**Scopus**<br>Searched via	TITLE-ABS-KEY (((labial AND frenum*) OR (labial AND frena) OR (labial AND frenulum) OR (labial AND frenula) OR (upper AND lip AND	Limited to: English language, Humans	

http://www.scopus.com/search/form.url?display=advanced&clear=t&origin=searchbasic&txGid=1lkb0B3HcbSzUk8cVtIzKL_%3a3 on 08.04.2023	frenum*) OR (upper AND lip AND frena) OR (upper AND lip AND frenulum*) OR (upper AND lip AND frenula) OR (upper AND frenum*) OR (upper AND frena) OR (upper AND frenulum) * OR (upper AND frenula) OR (maxillary AND frenum*) OR (maxillary AND frena) OR (maxillary AND frenulum*) OR (maxillary AND frenula) OR (maxillary AND labial AND frenum*) OR (maxillary AND labial AND frena) OR (mlf*)) AND ((diagnosis) OR (assessment*) OR (evaluation) OR (management) OR (frenotomy*) OR (frenectomy*) OR (referral*) OR (health AND impact))) AND (LIMIT-TO (LANGUAGE , "English")) AND (LIMIT-TO (EXACTKEYWORD , "Human") OR LIMIT-TO (EXACTKEYWORD , "Humans"))		
**ProQuest** Searched via http://proquest.com/advanced on 08.04.2023	summary(labial frenum* OR labial frena OR labial frenulum OR labial frenula OR upper lip frenum* OR upper lip frena OR upper lip frenulum* OR upper lip frenula OR upper frenum* OR upper frena OR upper frenulum* OR upper frenula OR maxillary frenum* OR maxillary frena OR maxillary frenulum* OR maxillary frenula OR maxillary labial frenum* OR maxillary labial frena OR MLF) AND summary(diagnosis OR assessment* OR evaluation OR management OR frenotomy* OR frenectomy* OR referral* OR health impact)	Searched by "Summary"	75
Google Scholar Beta Searched via www.scholar.google.com		Screened X first pages	
